# *In vitro* analysis of anti-HPA-1a dependent platelet phagocytosis and its inhibition using a new whole blood phagocytosis assay (WHOPPA)

**DOI:** 10.3389/fimmu.2023.1283704

**Published:** 2023-11-21

**Authors:** Paula Ames, Nelli Baal, Martin Speckmann, Gabriela Michel, Judith Ratke, Christina Klesser, Nina Cooper, Daisuke Takahashi, Behnaz Bayat, Gregor Bein, Sentot Santoso

**Affiliations:** ^1^ Institute for Clinical Immunology, Transfusion Medicine and Hemostasis, Justus Liebig University, Giessen, Germany; ^2^ Flow Cytometry Core Facility, Justus Liebig University, Giessen, Germany; ^3^ Research and Development, Japanese Red Cross, Tokyo, Japan

**Keywords:** platelets, FNAIT, HPA-1a, phagocytosis, monocytes, FcγR

## Abstract

Fetal and neonatal alloimmune thrombocytopenia (FNAIT) is a serious bleeding condition mostly caused by the reaction between maternal anti-HPA-1a antibodies and fetal platelets. This reaction leads to Fc-dependent platelet phagocytosis. Although several serological methods have been developed to identify maternal antibodies, a reliable laboratory parameter as a prognostic tool for FNAIT severity is still lacking. In this study, we developed whole blood platelet phagocytosis assay (WHOPPA), a flow cytometry-based phagocytosis assay that uses a pH-sensitive fluorescent dye (pHrodo-SE) to analyze anti-HPA-1a-dependent platelet phagocytosis in whole blood. WHOPPA revealed a high phagocytosis rate for the anti-HPA-1a opsonized platelets by monocytes but not by neutrophils. Analysis of different monocyte populations showed that all monocyte subsets, including classical (CD14^++^CD16^−^), intermediate (CD14^++^CD16^+^), and nonclassical (CD14^+^CD16^++^) monocytes, were able to engulf opsonized platelets. A unique monocyte subset, termed shifted monocytes (CD14^+^CD16^−^), showed the highest phagocytosis rate and was detected after platelet engulfment. FcγR inhibition tests revealed that except for FcγRIIa, FcγRI and FcγRIII on monocytes were responsible for the phagocytosis of anti-HPA-1a opsonized platelets. Analysis of anti-HPA-1a antibodies from FNAIT cases (n = 7) showed the phagocytosis of HPA-1aa but not of HPA-1bb platelets by monocytes. The phagocytosis rate was highly correlated with bound antibodies measured by flow cytometry (p < 0001; r = 0.9214) and MAIPA assay (p < 0.001; r = 0.7692). The phagocytosis rates were equal for type I and II anti-HPA-1a antibodies recognizing the plexin–semaphoring–integrin (PSI) domain and PSI/epidermal growth factor 1 domain of β3 integrin, respectively. By contrast, type III anti-HPA-1a antibodies reacting with αvβ3 integrin did not induce platelet phagocytosis. Furthermore, effector-silenced mAbs against HPA-1a inhibited the phagocytosis of anti-HPA-1a opsonized platelets. In conclusion, WHOPPA is a reliable *in vitro* platelet phagocytosis assay that mimics the phagocytosis of anti-HPA-1a opsonized platelets in whole blood. This assay allows to prove platelet phagocytosis *ex vivo* and evaluate the inhibitory capacity of different inhibitors as therapeutically strategies for the prevention of fetal thrombocytopenia in FNAIT in the future.

## Introduction

Fetal and neonatal alloimmune thrombocytopenia (FNAIT) is a serious bleeding condition caused by the transplacental transfer of maternal IgG alloantibodies reacting with platelet antigens expressed on fetal platelets. This antigen–antibody reaction leads to platelet phagocytosis, which is predominantly mediated by the interaction between the Fc part of bound antibodies and the Fcγ receptors (FcγRs) expressed on macrophages (1). In Caucasian population, anti-HPA-1a alloantibodies are responsible for about 85% of all FNAIT cases, with an incidence of approximately 1/1800 pregnancies (2). The clinical presentation of FNAIT varies from asymptomatic thrombocytopenia to severe clinical complications, including intrauterine growth restriction, intrauterine fetal demise, and intracranial hemorrhage (ICH). Among which, ICH is the most serious complication that occurs in around 10%–20% of FNAIT cases (3), leading to fetal death or persistent neurological sequelae in neonates (4). However, reliable laboratory parameters as a prognostic tool to identify fetuses/newborns at risk of severe FNAIT are still lacking (5).

HPA-1a is formed by a single amino acid substitution (Leu33Pro) located on the flexible plexin–semaphorin–integrin (PSI) domain of integrin β3 chain (6). On the platelet surface, the β3 chain can form heterodimers with either αIIb or αv subunit, functioning as the fibrinogen or vitronectin receptor, respectively (7). Recent studies demonstrated that immunized mothers could develop three different subtypes of anti-HPA-1a antibodies. The anti-HPA-1a subtypes I and II react with epitopes formed by the polymorphic PSI domain of the β3 chain alone and the epidermal growth factor 1 (EGF1) domain, respectively (8). Subtype III recognizes the antigenic determinants formed by αvβ3 heterodimer and could impair endothelial function associated with ICH in severe FNAIT cases (9). Whether subtype III antibody could also induce platelet phagocytosis is unclear.

From the current general view, maternal anti-HPA-1a antibodies bound to fetal platelets lead to fetal platelet clearance by monocytes/macrophages in the spleen (1). Recent evidence indicated that platelet antibodies could also cause platelet clearance via an Fc-independent mechanism, leading to platelet phagocytosis via the Ashwell–Morell receptor in the liver (10). In humans, three FcγRs, namely, FcγRI, FcγRII, and FcγRIII, are present during fetal development (11). The most abundant blood monocytes (classical subset) mainly express FcγRI and FcγRIIa, and a small population of monocyte subsets (nonclassical and intermediate subsets) express FcγRIIIa. Meanwhile, neutrophils express abundant FcγRIIIb (12). Determining which blood phagocyte subsets and which FcγRs are responsible for anti-HPA-1a-mediated platelet phagocytosis is necessary.

Our previous studies demonstrated that deglycosylated monoclonal antibody (mAb) against HPA-1a could abrogate platelet phagocytosis without interfering with FcRn-mediated placental transport (13). Whether such effector-silenced mAb could represent a novel inhibitor to prevent fetal platelet destruction by maternal anti-HPA-1a antibodies in FNAIT is worthy of exploration. Xu and colleagues (14) demonstrated that deglycosylated mAb GZ1 against CD36 could effectively prevent fetal death in an anti-CD36-mediated FNAIT mouse model. However, the efficacy of such approach in preventing anti-HPA-1a platelet clearance remains unclear.

In the last decades, a number of flow cytometry monocyte phagocytic assays have been developed (15). However, these methods may overestimate phagocytosis due to the nonspecific binding of fluorescence-labeled platelets to monocyte surface (16, 17). This problem could be overcome by using a pH-sensitive fluorescent dye (pHrodo) that only generates fluorescence signal when pHrodo-labeled platelets have entered lysosomes (acidic milieu) but not while they are in the cytoplasm or outside monocytes (18–20). However, this current technique is limited to the use of isolated phagocytic cells and therefore may not mimic the mechanism of antibody-mediated platelet phagocytosis in whole blood.

In this study, we developed a pHrodo-based platelet phagocytosis assay (PPA) using whole blood. Termed whole blood platelet phagocytosis assay (WHOPPA), this technique identifies the subset of phagocytic blood cells responsible for the phagocytosis of anti-HPA-1a opsonized platelets, to correlate the antibody titer with phagocytosis rate and to evaluate various inhibitors including anti-FcγRs and effector-silenced mAbs against HPA-1a.

## Materials and methods

### Antibodies

Anti-HPA-1a standard sera (03/152; human IgG1 and IgG2) were obtained from the NIBSC (Potters Bar Hertfordshire, UK). Anti-HPA-1a sera from FNAIT cases without anti-HLA (n = 7) were characterized in our platelet laboratory (21) (**Supplementary Table S1**).

This study was conducted in accordance with the Declaration of Helsinki and was approved by the Ethics Committee of the Medical Faculty, Justus Liebig University, Giessen, Germany (file no. 82/09 and file no. 05/00). Patient consent was waived due to the de-identified analysis of retrospective data or blood samples.

MAbs against CD64 IgG and F(ab’)2 fragment (murine IgG1; clone 10.1, BioLegend, Amsterdam, The Netherlands), CD32 (murine IgG2b, clone IV.3, StemCell, Cologne, Germany), CD16 IgG, and F(ab’)2 fragment (murine IgG1, clone 3G8, ThermoFisher, Dreieich, Germany) were used. Mouse IgG1κ (MOPS-21) and IgG2bκ (BPC4) isotype controls were obtained from BioLegend and Ancell (Hamburg, Germany), respectively. Fluorescence-labeled mAbs used for flow cytometry analysis were listed in the (**Supplementary Table S2**). Human mAbs against HPA-1a 26.4 (human IgG1), B2G1 (human IgG1), and B2G1 delta nab (human IgG1) were kindly provided by Dr. Tor B Stuge (Department of Medical Biology, University of Tromsø, Norway) and Dr. Winnie Lau (NHS Blood and Transplant, Cambridge, UK). Recombinant mAbs 26.4 IgG1 and IgG1 LALAP were produced by Biointron Biological Inc., Shanghai, China. Hybridoma producing mAb AP3 against β3 integrin (murine IgG1) was purchased from American Type Culture Collection (ATCC HB-242). Murine mAb against αvβ3 (murine IgG1, clone 23C6) and HPA-1a (murine IgG1; clone SZ21) were obtained from Millipore (Temecula, CA, USA) and Beckman Coulter (Marseille, France), respectively. IgG was purified using the Melon IgG kit (Thermo Scientific, Langenselbold, Germany), verified by 7.5% SDS-PAGE and silver staining using Prestained Protein Ladder (ThermoFisher), and adjusted to 10 mg/mL. Deglycosylated IgG was produced by the digestion of IgG-selective enzyme endoglycosidase S (EndoS, New England Biolabs, Frankfurt, Germany) and characterized as previously described (14).

### WHOPPA

Isolation, labeling of platelets with pHrodo, and sensitization by antibodies were described in the supplementary data. Aliquots (200 µL) of sensitized, pHrodo-stained platelets (1.5 x 10^7^ platelets) were incubated with 200 µL of whole blood containing 5 x 10^4^ monocytes for 60 min at 37°C and 5% CO_2_. After the erythrocytes in whole blood were lysed with lysis buffer (155 mM ammonium chloride, 10 mM potassium hydrogen carbonate, and 100 µM EDTA) for 5 min, the monocytes were labeled with 3 µL of APC-conjugated anti-CD14 mAb for 30 min in the dark. Viable monocytes were identified by flow cytometry after staining with 1 µL of SYTOX Blue (Invitrogen, Paisley, UK). All CD14^+^ and pHrodo^+^ cells were defined as viable monocytes that phagocytosed sensitized platelets. Approximately 5,000–20,000 cells were evaluated. The phagocytosis rate was defined as the percentage of monocytes that engulfed platelets. In some experiments, the neutrophils were labeled with 3 µL of Pacific Blue-conjugated anti-CD16 mAb to examine platelet engulfment by neutrophils. Whole blood was incubated with 20 μg of pHrodo-stained *E. coli* bacteria as control (Life Technologies Corporation, Eugene, USA) at 4°C and 37°C and analyzed by flow cytometry using BD FACSCanto™ II Flow Cytometer and BD FACSAria™ III Cell Sorter and BD FACSDiva™ software v6.1.3 (Becton Dickinson Biosciences). To exclude false negative gating of neutrophils due to shedding of CD16 antigen from neutrophil surface, additional gating strategy independently from CD16 marker was performed to identify neutrophils and monocytes (see below).

### Analysis of neutrophil and monocyte subsets

The neutrophils and monocytes were analyzed by flow cytometry using gating strategy as previously described (22). Leukocyte subsets derived from whole blood were separated by side scatter (SSC) and CD45 marker. The monocytes and neutrophils were identified as SSC low and SSC high, respectively. Cell aggregates (“doublets”) and other nontarget subsets (T-, B-, dendritic, and dead cells) were excluded by forward scatter area (FSC-A) against forward scatter height and by negative selection using CD1c, CD3, CD19, and live/death markers (lineage cocktail), respectively. Finally, the viable neutrophils (CD66b^+^) and monocytes (CD66b^−^) were analyzed for pHrodo signal, FcγRI, FcγRII, FcγRIII, and HLA-DR expression. The monocyte subsets were gated with anti-CD14 (clone M5E2) and anti-CD16 (clone LNK16) as described (23).

### t-SNE analysis of monocyte subsets

T-distributed stochastic neighbor embedding (t-SNE) analysis was performed as previously described (24). Individual fcs files were analyzed by FlowJo (FlowJo™ v10.9 Software; BD Life Sciences). The viable monocytes were gated as described before (**Supplementary Figure S3**) and cleaned of time artifacts before the population size was normalized to 50.000 cells using the FlowJo plugin DownSample v3.3.1 (BD life Science). The fcs files were then concatenated into a single fcs file with a total of 100.000 cells to create a t-SNE map. Barnes–Hut implementation of t-SNE was used to apply the dimensionality reduction algorithm (25). Best separation of monocyte subsets was achieved by generating a global t-SNE map using CD64, CD32, and CD16 markers under the following running conditions (iterations: 5.000 iterations; perplexity parameter: 90; and learning rate: 9.000). t-SNE maps were generated by plotting t-SNE (x- and y-axis) in a dot plot. Color mapping was then applied by overlaying the fluorescence intensity for additional markers (CD14, HLA-DR, and pHrodo) to assess unknown map clusters. Within the heatmap overlays, the scaling of all markers was optimized uniformly, ranging from low (−1635.8786, blue) to high signal (84005.4883; red). In addition to the pHrodo heatmap, the overlays of pHrodo positive and high positive subsets were performed to evaluate map regions positive (compared with negative control) or highly positive (top 1% with highest fluorescence intensity) for pHrodo signals. Previously gated monocyte subsets were overlaid onto the t-SNE dot plot for subset phenotype comparison based on t-SNE map position.

### Analysis of platelet antibody binding by flow cytometry

A 10 µL aliquot of sensitized pHrodo-labeled platelets in HBSS buffer was incubated with 1 µL of FITC-conjugated rabbit F(ab)_2_ antihuman IgG (dilution 1:10; Dako, Glostrup, Denmark) for 15 min in the dark and analyzed by flow cytometry. The platelets were gated in forward/side scatter (FSC/SSC). Antibody binding was calculated based on the median fluorescence intensity (MFI).

### Inhibition of platelet phagocytosis by effector silencing anti-HPA-1a and anti-FcγR antibodies

PHrodo-stained platelets were incubated with 10 µL of effector silencing anti-HPA-1a antibodies (8 µg) at 37°C before incubation with anti-HPA-1a antibodies. Deglycosylated IgG from isotype control or AB serum was run in parallel. For FcγR inhibition, whole blood or isolated monocytes were incubated with anti-CD64, anti-CD32, anti-CD16, isotype control (10 µg/ml) IgG, or an equal amount of F(ab’)2 fragment for 30 min at room temperature before phagocytosis.

## Results

### Monocytes, not neutrophils, engulf anti-HPA-1a-opsonized platelets in whole blood

**Figure 1:**
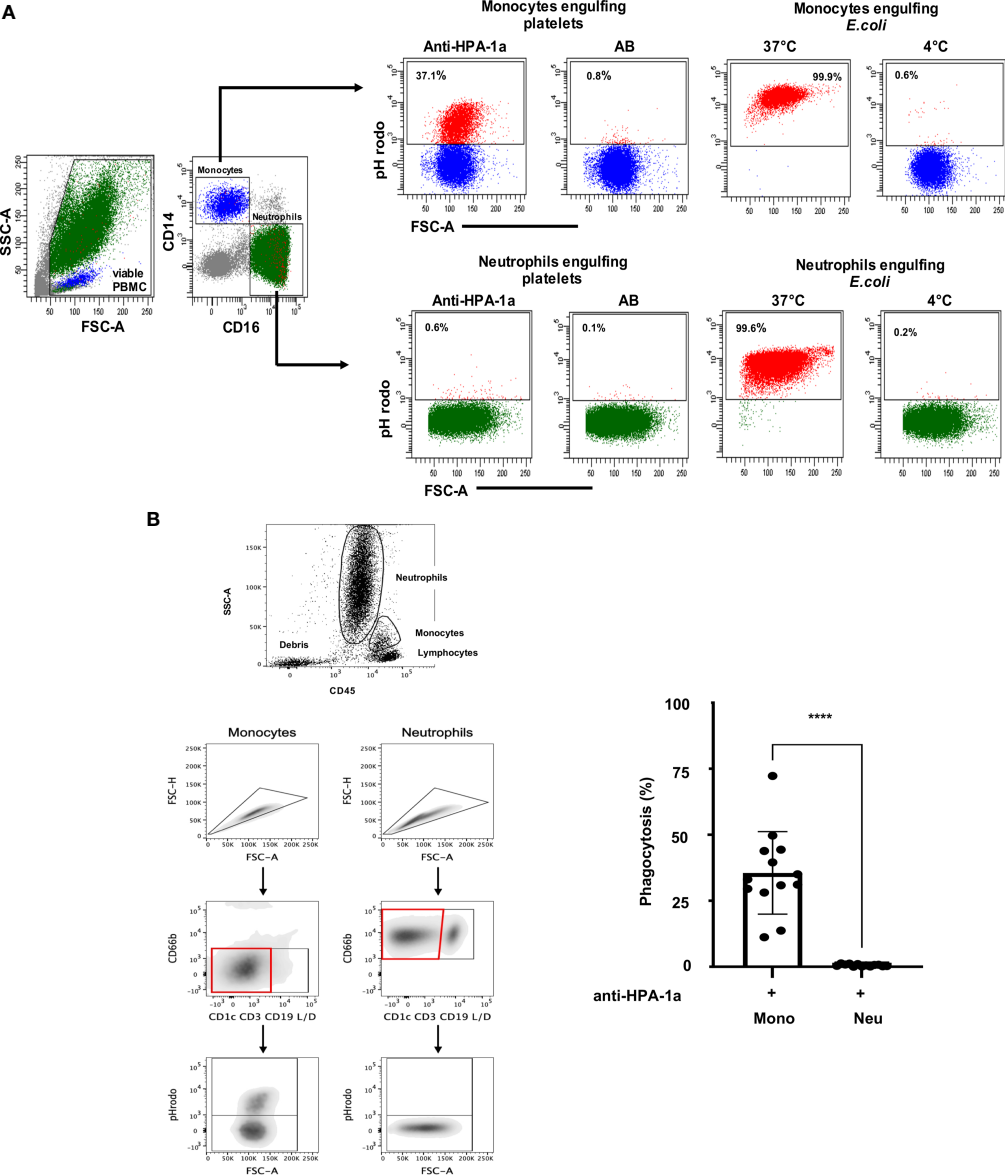
**(A)** Anti-HPA-1a mediated platelet phagocytosis by monocytes and neutrophils. PHrodo-labeled platelets were sensitized with anti-HPA-1a standard serum (dilution 1:16) or AB serum (as control) and subjected to WHOPPA. Peripheral blood mononuclear cells (PBMCs) were gated by forward scatter (FSC-A) and side scatter (SSC-A). Viable cells were identified using SYTOX Blue staining. Total monocytes (blue) and neutrophils (green) were identified by staining with APC-conjugated anti-CD14 and Brilliant Violet 510-conjugated anti-CD16, respectively. The percentages of monocytes that engulfed platelets (pHrodo^+^) are indicated. In the control experiments, pHrodo-labeled *E*. *coli* bacteria were subjected to phagocytosis by monocytes and neutrophils at 37°C and 0°C (negative control). **(B)** Anti-HPA-1a mediated platelet phagocytosis by monocytes and neutrophils *Left panel* (Gating strategy): Cell aggregates (“doublets”) and other nontarget subsets (T-, B-, dendritic, and dead cells) were excluded by forward scatter area (FSC-A) against forward scatter height (FSC-H) and by negative selection using CD1c, CD3, CD19, and live/death markers (lineage cocktail), respectively. The viable neutrophils (CD66b^+^) and monocytes (CD66b^−^) were analyzed for pHrodo signal and FcγRI, FcγRII, FcγRIII and HLA-DR expression. *Right panel*: PHrodo-labeled platelets were sensitized with anti-HPA-1a standard serum (dilution 1:16) or AB serum (as control) and subjected to WHOPPA. The percentage of phagocytic monocytes (Mono) and neutrophils (Neu) derived from 13 biological replicates is presented. Bars indicate means ± SEM. Statistical significance was assessed by one-way ANOVA with Tukey posthoc test. ***p < 0.001.

A new fluorescence-based PPA using pH-sensitive fluorescent dye (pHrodo) was established to analyze anti-HPA-1a-mediated platelet phagocytosis in whole blood. The flowchart of WHOPPA is illustrated in (**Supplementary Figure S1**). Viable monocytes and neutrophils were defined by labeling with APC- or Pacific Blue-A-conjugated mAbs against CD14 or CD16, respectively (**Figure 1A**). The capability of two major blood phagocytes, namely, monocytes and neutrophils, to engulf anti-HPA-1a (03/152; dilution 1:10) opsonized platelets (pHrodo^+^) was determined by flow cytometry. Only monocytes (37.1%), not neutrophils (0.6%), engulfed the anti-HPA-1a-opsonized platelets in whole blood. In the control experiments, only 0.6% monocytes and 0.1% neutrophils engulfed the platelets opsonized with AB serum. By contrast, a comparably high amount of pHrodo-labeled *E. coli* was engulfed by monocytes and neutrophils (99.9% and 99.6%) at 37°C. Only 0.6% monocytes and 0.2% neutrophils could engulf *E. coli* bacteria at 4°C.

Since neutrophils could shed CD16 from the cell surface after platelet phagocytosis leading to false negative determination of engulfed monocytes, additional gating strategy independently from CD16 marker (**Figure 1B**; left panel)) was conducted. Analysis of different blood donors (n=13) showed the engulfment of opsonized platelets by monocytes but not by neutrophils in whole blood (**Figure 1B**, right panel).

Furthermore, the specificity of platelet phagocytosis by monocytes was supported by confocal and electron microscopy analysis (**Figure S2**).

**Figure 2:**
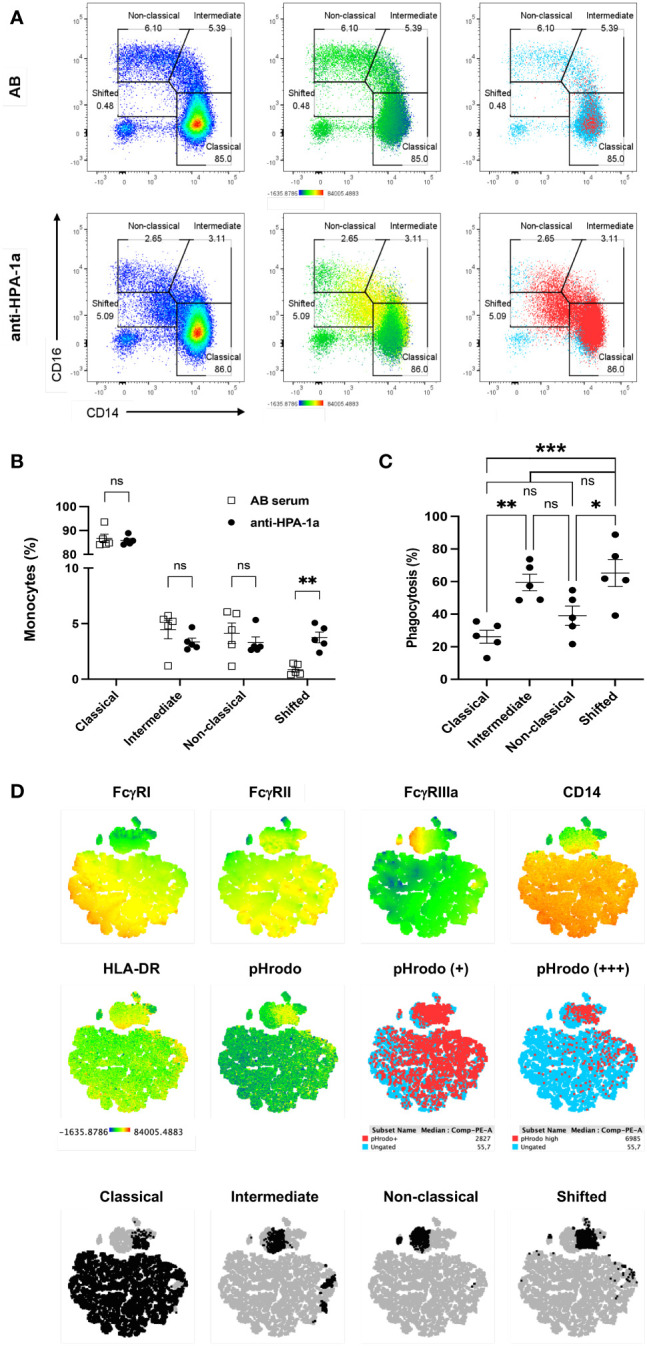
Anti-HPA-1a mediated platelet phagocytosis by monocyte subsets. PHrodo-labeled HPA-1aa-typed platelets were opsonized with AB serum (control) and anti-HPA-1a standard serum and subjected to WHOPPA as described above. Monocyte subsets were identified according to the gating strategy described by Fujimoto et al., 2000 (22). **(A)** Monocytes were gated for classical (CD14^++^CD16^-^), intermediate (CD14^++^CD16^+^), nonclassical (CD14^+^CD16^++^), and shifted (CD14^+^CD16^-^) monocyte subsets. Left panels: cell density shown by a heatmap color gradient from low to high (blue-red). Middle panels: overlay of pHrodo signal intensity displayed by a heatmap color gradient from no signal to high signal (blue to red). Right panels: overlay of pHrodo positive monocytes (red). Ungated monocytes are indicated by blue dots. **(B)** Percentage of classical, intermediate, nonclassical, and shifted monocyte subsets upon the phagocytosis of anti-HPA-1a (black circles) and AB serum-opsonized platelets (white squares). **(C)** Percentage of monocyte subsets after the phagocytosis of anti-HPA-1a opsonized platelets. The results of five biological replicates are presented; bars indicate means ± SEM. Statistical significance was assessed by one-way ANOVA with Tukey posthoc test. *p < 0.05, **p < 0.01; Cut-off of phagocytosis is 2.5%; ns, not significant, **(D)** t-SNE-guided phenotype comparison of monocyte subsets. After the engulfment of platelets opsonized with AB serum or anti-HPA-1a, monocytes from one representative donor were gated and merged to create a single t-SNE map of 100.000 events. Dimensionality reduction analysis by t-SNE was performed as described in *Materials and Methods*. **(A)** Signal intensity of CD64 (FcγRI), CD32 (FcγRII), CD16 (FcγRIIIa), CD14, HLA-DR, and pHrodo projected onto the t-SNE space according to a heatmap color scale ranging from low signal (blue) to (high signal, red), **(B)** positive and high positive for pHrodo signal (red), and **(C)** Manually gated monocyte subsets (black) were overlaid onto the t-SNE map and highlighted by specific colors. Ungated subsets (light blue or gray) refer to all events.

Subsequently, monocytes were gated (**Figure S3**), and their subsets were analyzed in detail to study the role of different monocyte subsets on anti-HPA-1a-mediated platelet phagocytosis (**Figure 2A**; see also **Figure S4**). Three well-known monocyte subsets, namely, classical (CD14^++^CD16^−^), intermediate (CD14^++^CD16^+^), and nonclassical (CD14^+^CD16^++^) monocytes, were detected when the platelets were opsonized with AB serum (control). A small unique monocyte subset (CD14^+^CD16^−^) termed “shifted” monocytes was found solely after the engulfment of anti-HPA-1a opsonized platelets. This result could be confirmed by repeated experiments with different donors (**Figure 2B**). As shown in **Figure 2C**, all three monocyte subsets were capable of mediating platelet phagocytosis. Although classical monocytes (CD14^++^CD16^−^) were the most abundant monocyte subset in whole blood, they showed significant lower phagocytosis capability than the intermediate (CD14^++^CD16^+^). On the basis of the FcγR expression pattern, the shifted monocytes were most likely derived from all three monocyte nsubsets (**Figure 2D**).

**Figure 3:**
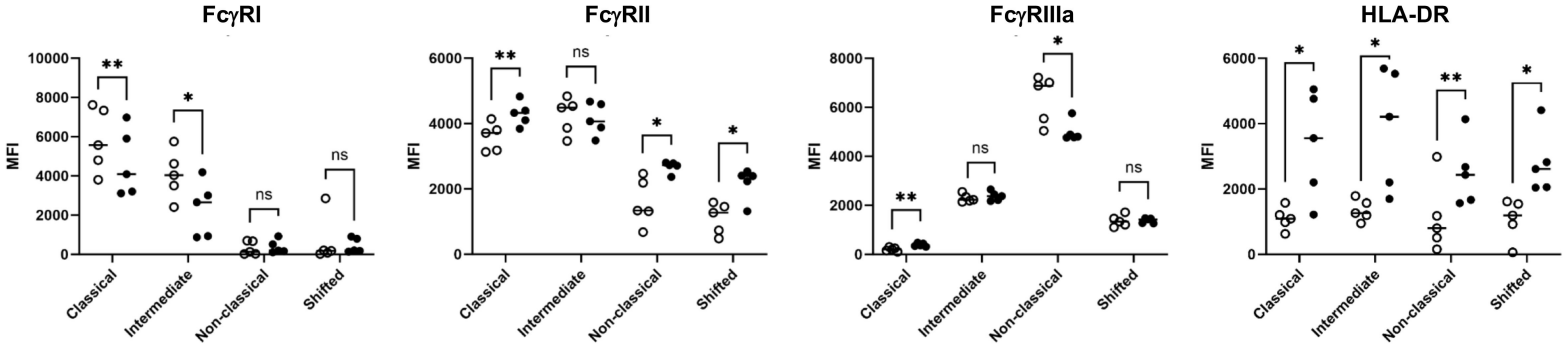
FcγR and HLA class II expression of monocyte subsets. After the engulfment of anti-HPA-1a opsonized platelets, monocyte subsets that engulfed anti-HPA-1a opsonized platelets were gated for pHrodo^−^ (open circle) and pHrodo^+^ populations (closed circle) and analyzed for FcγRs and HLA-DR as indicated. The results of five biological replicates are presented. Median fluorescence intensity (MFI) was normalized by isotype or negative controls. Data distribution was analyzed using Shapiro–Wilk and Kolmogorov–Smirnov tests. Paired two-tailed two-sample t-tests were performed, and significance levels were indicated as * (p ≤ 0.05), ** (p ≤ 0.01), and *** (p ≤ 0.001).

Furthermore, we analyzed the expression of FcγRs and HLA-DR on different monocyte subsets (**Figure 3**). FcγRI (CD64) expression was found on classical and intermediate subsets but not on nonclassical and shifted subsets. The significantly reduced CD64 expression after the phagocytosis of anti-HPA-1a-opsonized platelets indicated the role of ligand-induced internalization mediated by CD64 receptor. A similar phenomenon was observed for FcγRIIIa (26). By contrast, FcγRII was detected in all monocyte subsets and was upregulated after platelet phagocytosis. Similarly, HLA-DR was upregulated in all monocyte subsets that had engulfed opsonized platelets, and this phenomenon was most probably associated with monocyte activation (27). Overall, these results suggested that FcγRI and FcγRIIIa are involved in platelet phagocytosis.

### Anti-FcγRI and anti-FcγRIII antibodies inhibited the phagocytosis of anti-HPA-1a opsonized platelets in whole blood

**Figure 4:**
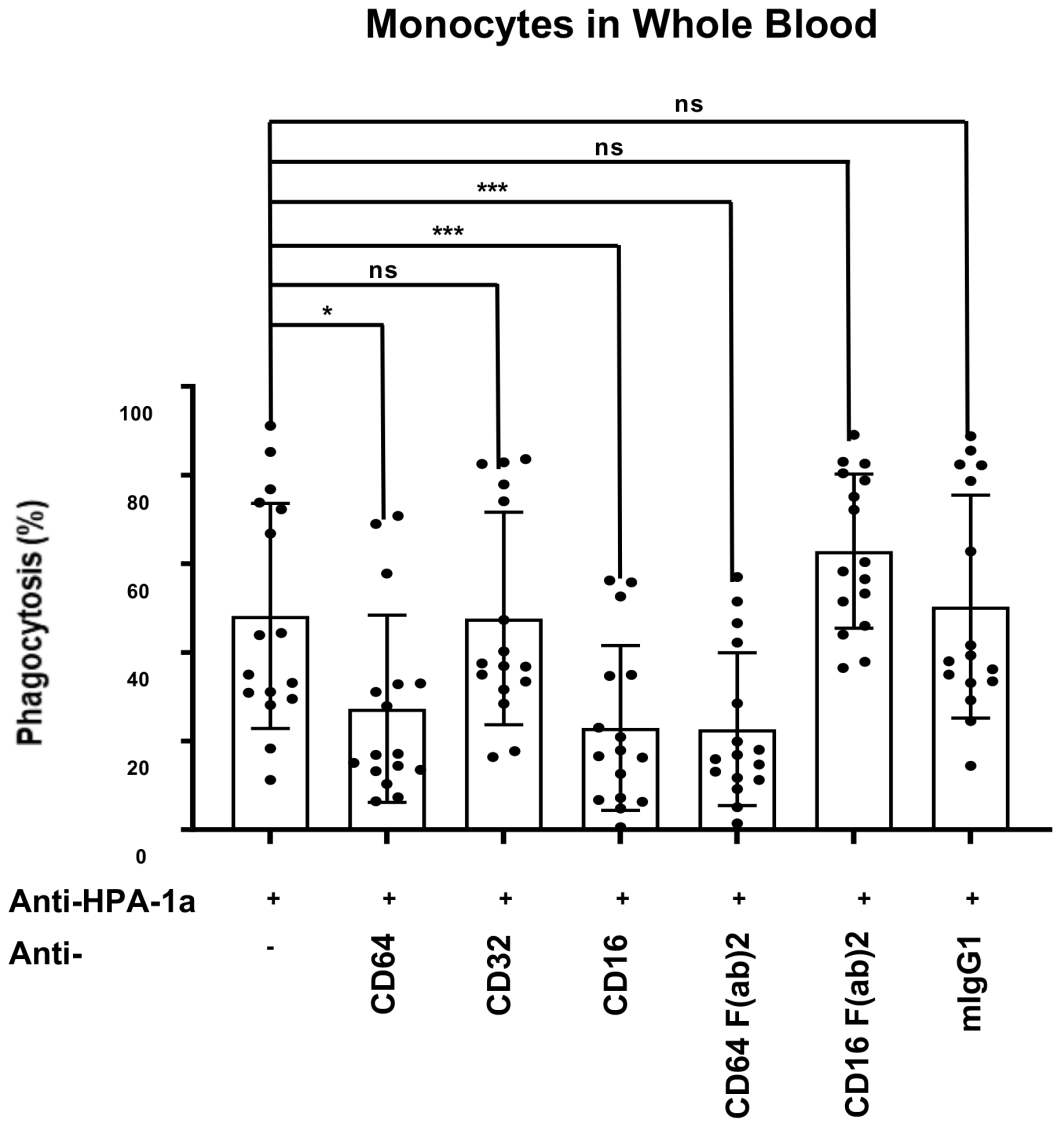
Inhibition of anti-HPA-1a-mediated platelet phagocytosis by mAbs against FcγRs. PHrodo-labeled HPA-1aa-platelets (n = 16) were sensitized with anti-HPA-1a standard serum (dilution 1:16) and incubated with whole blood pretreated with mAbs against FcγRI, FcγRII, and FcγRIII IgG or F(ab’)2 fragment. Isotype mouse IgG1κ was used as a negative control. The platelet phagocytosis rate (%) in whole blood was analyzed. Friedmann Test was performed, and significant level was set as *p ≦ 0.05, **p ≦ 0.01, ***p ≦ 0.001, and ****p ≦ 0.0001. Error bars represent means ± SD; ns, not significant.

Monocytes in whole blood were incubated with mAbs specific for anti-FcγRI (CD64), FcγRII (CD32), and anti-FcγRIII (CD16) and analyzed by WHOPPA to further evaluate the role of different FcγRs on anti-HPA-1a mediated phagocytosis (**Figure 4**). In accordance to the previous observation, the suppression of FcγRI and FcγRIII with anti-CD16 and anti-CD64 IgG antibodies significantly inhibited platelet phagocytosis when compared to isotype control. By contrast, anti-CD32 IgG did not block platelet phagocytosis. Analysis with F(ab’)2 fragments showed that anti-CD16 F(ab’)2 fragment did not inhibit platelet phagocytosis (IgG versus F(ab’)2 p<0.001), whereas anti-CD64 F(ab’)_2_ still did (IgG versus F(ab’)2 not significant). Although CD16 is expressed only less than 20% of monocytes, anti-CD16 IgG antibodies were able to reduce the phagocytosis rate by around half. This observation suggested that anti-CD16 could not only directly impair monocytes via Fab part (cis-effect), but also indirectly (trans-effect) via Fc part.

### WHOPPA revealed a high correlation between bound antibodies and phagocytosis

**Figure 5:**
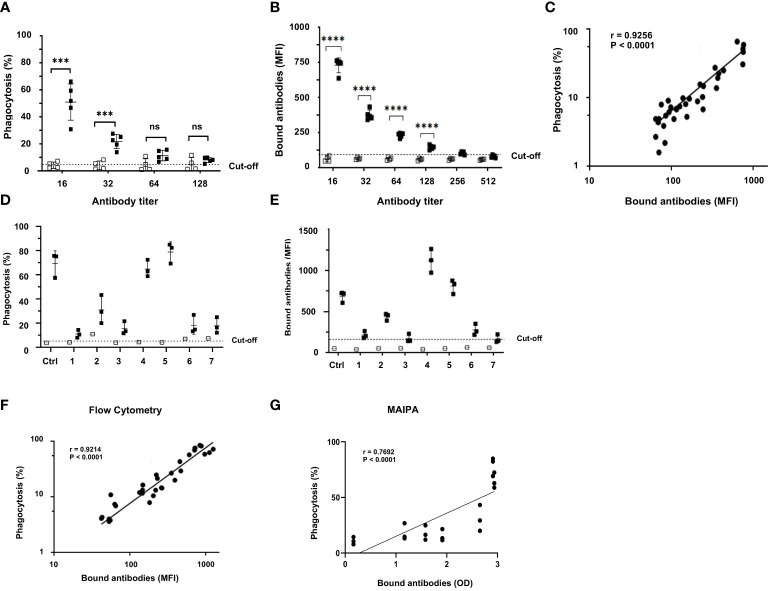
Correlation between anti-HPA-1a antibody titer and phagocytosis rate. **(A)** PHrodo-labeled HPA-1aa (black; n = 5) and HPA-1bb (white; n = 4) platelets were incubated with standard anti-HPA-1a sera (NIBSC 03/152; dilution 1:16-1:512). The opsonized platelets were then subjected to WHOPPA, **(B)** In parallel, platelet-bound anti-HPA-1a antibodies were measured by flow cytometry using fluorescence-labeled rabbit antihuman F(ab’)_2_ fragment, **(C)** The correlation between bound antibodies (MFI) and phagocytosis rate (%) is shown. The cut-off of antibody binding is defined as the MFI of normal IgG plus 2SD, **(D)** PHrodo-labeled HPA-1aa (black; n = 3) and HPA-1bb (white; n = 1) platelets were incubated with FNAIT sera (n = 7) containing anti-HPA-1a (final dilution 1:10) and then subjected to WHOPPA, **(E)** In parallel, the corresponding bound anti-HPA-1a antibodies was measured by flow cytometry, **(F)** between bound antibodies (MFI) measured by flow cytometry and phagocytosis rate (%) and **(G)** measured by the antigen capture assay (MAIPA; OD) and phagocytosis rate (%) are shown. The cut-off of antibody binding is defined as the MFI of normal IgG plus 2SD. *** *p* ≦ 0.001, and ****p ≦ 0.0001. ns: not significant.

Homozygous HPA-1a (+) and HPA-1a (−) typed platelets were sensitized with different dilutions of anti-HPA-1a standard serum (NIBSC 03/152; 1:16–1:512 dilutions) to further evaluate the specificity and sensitivity of anti-HPA-1a-mediated platelet phagocytosis by monocytes. Significant platelet phagocytosis was observed only with sensitized HPA-1a (+) but not with HPA-1a (−) platelets (**Figure 5A**), which remained with 1:32 diluted serum (**Figure 5B**). Bound anti-HPA-1a was still observed with 1:128 diluted sera, indicating that a certain threshold of antibody level is necessary to trigger platelet phagocytosis. Nonetheless, a significant correlation (r = 0.9256; *p* < 0.0001) was observed between antibody binding (MFI) measured by flow cytometry and phagocytosis rate (**Figure 5C**). All tested anti-HPA-1a samples (n = 7) induced allele-specific phagocytosis, but only HPA-1a (+), not HPA-1a (−), opsonized the platelets (**Figure 5D**). The phagocytosis rate was correlated with the quantity of platelet-bound antibodies (r = 0.9214, *p* < 0.0001) (**Figures 5E, F**). However, a low correlation was observed when antigen capture assay, MAIPA, was used to measure bound antibodies (**Figure 5G**).

### Anti-HPA-1a antibody subtypes I and II, not III, caused platelet clearance

**Figure 6:**
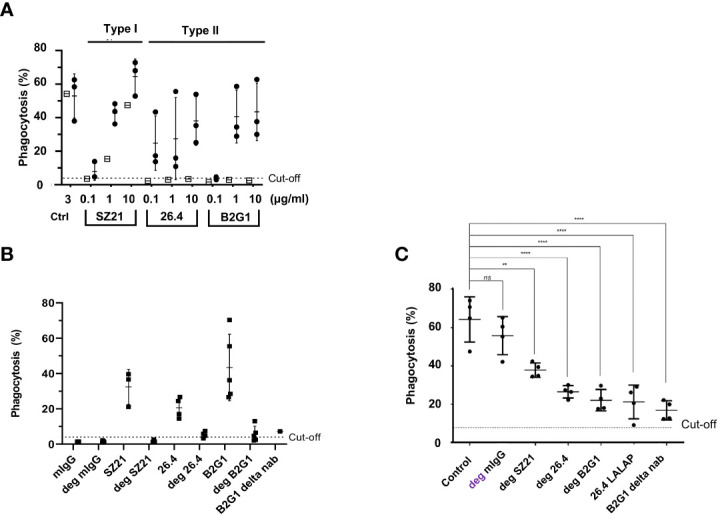
Analysis of type I and type II and effector silencing anti-HPA-1a in WHOPPA. **(A)** PHrodo-labeled HPA-1aa (n = 3; black circles) and HPA-1bb (n = 1; white squares) platelets were sensitized with different concentrations (0.1, 1, and 10 µg/ml) of anti-HPA-1a mAbs as indicated. In the control experiment, platelets were opsonized with isotype controls (Ctrl). **(B)** PHrodo-labeled HPA-1aa platelets (n = 4) were sensitized with either native or deglycosylated (deg) mAbs against HPA-1a as indicated. **(C)** pHrodo-labeled HPA-1aa platelets were opsonized with anti-HPA-1a standard serum (dilution 1:16) alone or with deg-mAb SZ21, 26.4, and B2G1 or effector silencing 26.4 LALAP or B2G1 delta nab. The platelet phagocytosis rate (%) was analyzed by WHOPPA. **p< 0.01 and ***p< 0.001, ns, not significant. Normal mouse IgG and deglycosylated IgG (mIgG; deg-mIgG) were run in parallel as controls.

Two different subtypes (I and II) of anti-HPA-1a antibodies reacting solely with the polymorphic β3 subunit have been reported. Subtype I anti-HPA-1a antibodies recognize HPA-1a epitopes residing on the PSI domain, and subtype II antibodies react with complex epitopes formed by the PSI together with the EGF1 domain of β3 integrin (8, 28). As shown in **Figure 6A**, subtypes I (mAb SZ21) and II (mAbs B2G1 and 26.4) induced the phagocytosis of HPA-1a (+) but not HPA-1a (−) platelets. However, subtype I mAb SZ21 at a high concentration (10 μg/mL) also induced the phagocytosis of HPA-1a (−) platelets. This phenomenon is in accordance with our previous real-time antibody binding analysis using surface plasmon resonance technology, demonstrating that mAb SZ21 could also interact with immobilized HPA-1b antigen. However, this interaction was 100-fold weaker than that with HPA-1a antigen (13).

Preliminary experiment showed that type III anti-HPA-1a antibodies did not inhibit platelet phagocytosis, most probably due to low expression of αvβ3 on platelet surface (data not shown).

### Silenced mAbs against HPA-1a inhibited anti-HPA-1a-mediated platelet phagocytosis

Removing N-glycan (linked to asparagine 297) on the Fc part significantly weakens the binding of IgG to FcγRs and its ability to activate complement factor C1q (29). IgG antibodies were digested by EndoS to test the phagocytosis capability of deglycosylated mAbs against HPA-1a, and only a completely digested IgG fraction was used in this study (**Figure S5**). In contrast to naïve IgG, all deglycosylated mAbs (SZ21, 26.4, and B2G1) samples against HPA-1a were unable to induce platelet phagocytosis (**Figure 6B**). In the control experiment, Fc-silenced B2G1 delta nab did not induce platelet phagocytosis. Inhibition experiments were then performed to investigate whether Fc-silenced anti-HPA-1a mAbs could block platelet phagocytosis induced by anti-HPA-1a antibodies. Deglycosylated mAbs SZ21, 26.4, and B2G1 and B2G1 delta nab significantly inhibited anti-HPA-1a-mediated platelet phagocytosis. A similar result was obtained with another Fc-silenced anti-HPA-1a mAb (26.4 IgG1 LALAP) (**Figure 6C**).

## Discussion

Fetal bleeding in FNAIT cases is mostly attributed to maternal platelet alloantibodies, which opsonize fetal platelets and thereby enhance platelet clearance by FcγR-mediated phagocytosis in the reticuloendothelial system of the spleen (1). Different alloantibodies against HPAs associated with FNAIT are reliably detected by serological methods. However, the ability of these antibodies to trigger platelet clearance appears to be heterogeneous, and the mechanism underlying this phenomenon is not completely understood. Therefore, an *in vitro* PPA that mimics *in vivo* conditions must be developed to improve our understanding regarding the clinical relevance of platelet antibody-induced thrombocytopenia.

In the last decades, a number of monocyte monolayer fluorescence-based PPAs have been developed (16, 17). Among them, the most reliable method is PPA using pHrodo-labeled platelets that only generates fluorescence signal in acidic lysosomes milieu of phagocytosing cells (18–20). However, such monocyte monolayer approach is difficult to standardize (15) and does not allow for the simultaneous analysis of blood phagocytes. Here, we developed WHOPPA that abrogates these drawbacks, allowing for the specific analysis of engulfed platelets under standardized conditions that nearly mimic *in vivo* environment.

Given that WHOPPA uses whole blood, opsonized platelets could theoretically be phagocytosed not only by monocytes but also by other blood phagocytes, such as neutrophils. Here, we found that only monocytes, but not neutrophils, could engulf anti-HPA-1a-sensitized platelets. Although monocytes and neutrophils share many properties, they have distinctive morphologic and functional characteristics, including granules protein production, chemotactic response, and metabolic burst activity during phagocytosis (30). Furthermore, monocytes constitutively express FcγRI in contrast to neutrophils, which only express this receptor in response to inflammatory stimuli (30). The fact that neutrophils were capable to engulf platelets in both antibody and non-antibody mediated manner has been documented. Previous studies, however, were performed with either isolated monocytes (18, 19) or with activated platelets (31, 32), but not with whole blood and non-activated platelets as in the experimental setting of WHOPPA. Actually, the reason for this discrepancy is not yet clear.

Monocytes are currently divided in three subsets according to the relative surface expression of CD14 and CD16. The classical subset represents the most abundant portion (80%) of the total monocyte population, and the remaining 20% express CD16 and are further classified into nonclassical and intermediate subsets (33). Classical monocytes are phagocytic without inflammatory attributes, and nonclassical monocytes display inflammatory characteristics and exhibit properties for antigen presentation. Intermediate monocytes are a minor transitional subset that displays phagocytic and inflammatory functions (34, 35). In this study, we found that all three monocyte subsets can engulf anti-HPA-1a opsonized platelets. Although classical monocytes are the most abundant monocyte population in whole blood, they showed significant lower phagocytosis capability than intermediate and nonclassical subsets, indicating the importance of inflammatory response attributes in anti-HPA-1a-mediated platelet phagocytosis. A new monocyte subset (termed “shifted” subset) appeared after platelet phagocytosis most probably due to the loss of CD14 and CD16 expression after engulfment of platelets.

Furthermore, we detected the significant inhibition of platelet phagocytosis with anti-FcγRI and anti-FcγRIII but not with anti-FcγRII antibodies. The inhibitory effect of anti-FcγRIII F(ab’)2 disappeared when F(ab’)2 fragment was applied. Meanwhile, anti-FcγRI F(ab’)2 remained inhibitory. Only anti-FcγRIII as intact IgG strongly inhibited platelet phagocytosis in whole blood, indicating the involvement of other FcγR-carrying blood cells. Wiener et al. (17, 36) demonstrated the significant inhibition of anti-HPA-1a opsonized platelet phagocytosis by isolated monocytes with humanized mAb H22 specific for FcγRI but not with anti-FcγRIII (mAb 3G8). Furthermore, previous clinical studies showed that treatment with mouse and humanized anti-FcγRIII (mAb 3G8 and GMA161) for a patient with refractory immune thrombocytopenic purpura led to increased platelet counts, accompanied by neutropenia (37, 38). These phenomena might be a result of apoptotic neutrophil clearance by macrophages due to the binding of anti-CD16 antibodies to FcγRIIIb expressed on neutrophils (39).

Our result is in accordance with a recent study demonstrating the importance of FcγRI and FcγRIII on the phagocytosis of platelets opsonized with anti-αIIbβ3 autoantibodies from patient with immune thrombocytopenia by splenic macrophages (40). The less important role of FcγRII on anti-HPA-1a-mediated platelet phagocytosis was also indicated in an earlier work (17). Rijkers and coworkers showed that the cross-linking of FcγRIIa on platelets by antibodies (anti-HLA antibodies) could trigger platelet activation and subsequent platelet phagocytosis by macrophages (41). Whether certain anti-HPA-1a antibodies could also induce phagocytosis via this pathway remains unknown. When maternal anti-HPA-1a from different FNAIT cases were tested by WHOPPA, a strong correlation between phagocytosis rate and bound anti-HPA-1a was observed when antibody binding was measured by flow cytometry and MAIPA. However, a relatively low correlation was observed with MAIPA. This difference is attributed to the different detection systems: fluorescence- (linear reaction) versus enzyme- (nonlinear reaction) based assay. Titration analysis of standard anti-HPA-1a serum showed that platelet phagocytosis was no longer present at an antibody titer of 1:64, although the binding of anti-HPA-1a antibody was still measurable by flow cytometry. This observation revealed that a certain minimum number of antibody-antigen interaction is required to initiate platelet phagocytosis and served as an indication why platelet phagocytosis may not occur for platelets carrying low copy number of antigen, such as HPA-5 (42).

Previous clinical studies, however, showed that the titer of anti-HPA-1a antibodies was not strictly correlated with fetal thrombocytopenia (43), indicating that other confounding factors such as antibody fucosylation (18) and CRP (19) play also important role. Besides this aspect, the question of whether the quantification of *in vitro* phagocytosis of anti-HPA-1a opsonized platelets by WHOPPA correlates with clinical outcomes, such as fetal or neonatal platelet count in anti-HPA-immunized mothers is another limitation of this study, that should be addressed in the near future.

Our previous study showed that the effector silencing of deglycosylated mAb SZ21 against HPA-1a (deg-SZ21) could prevent platelet phagocytosis by macrophages in the NOD/SCID mouse model (13). However, recent investigations showed that anti-HPA-1a epitopes are heterogeneous, which is expected for a polyclonal immune response (8, 9). Therefore, a panel of anti-HPA-1a mAbs recognizing different epitopes is required to displace the binding of maternal anti-HPA-1a antibodies to prevent fetal platelet phagocytosis from the circulation. Using WHOPPA, we showed that both types (type I, mAb SZ21 and type II, mAbs 26.4; B2G1) of effector-silenced mAbs against HPA-1a did not induce platelet phagocytosis but could prevent platelet anti-HPA-1a-mediated platelet phagocytosis. However, complete inhibition could not be achieved by each of these antibodies alone or in combination, indicating that a selected panel of mAbs is necessary to inhibit the binding of maternal anti-HPA-1a antibodies.

Lastly, whether IgG-mediated platelet phagocytosis solely occurrs in the spleen remains unclear. Biburger and colleagues reported that splenectomized mice were still capable of depleting platelets, suggesting that splenic-resident phagocytic cell populations are not required for platelet depletion (44). Through this mouse model, the authors also demonstrated the nonrequirement of neutrophils and underlined the importance of monocyte subsets for IgG-dependent platelet depletion.

We established a simple, reliable, and specific *in vitro* assay that mimics platelet phagocytosis by monocytes caused by anti-HPA-1a antibodies in whole blood. This assay allows us to study the mechanism of antibody-mediated platelet phagocytosis under *ex vivo* conditions and evaluate the inhibitory capacity of different inhibitors as therapeutically strategies for the prevention of fetal thrombocytopenia in FNAIT in the future.

## Methods

### Platelet isolation

Platelets from HPA-1 (a+b−) and HPA-1 (a−b+) individuals were isolated and labeled with pHrodo Red succinimidyl ester (pHrodo, Life Technologies, Darmstadt, Germany) as previously described.^19^ Platelet-rich plasma (PRP) was isolated from 10 mL of acid citrate dextrose (ACD)-anticoagulated blood by centrifugation (120 g, 25 min). After being washed (10 min, 800 g) with phosphate-buffered saline (PBS) (Dulbecco, Anprotec, Bruckberg, Germany) containing 10 mM EDTA and 0.3 µM prostaglandin E1 (PEP, pH 7.4), the platelets were adjusted to a concentration of 7.5 x 10^8^/mL with PEP buffer. A 540 µL aliquot of platelet suspension (7.5 x 10^8^/mL) was incubated with 60 µL of pHrodo (final concentration 3.3 µM) for 30 min in the dark. The labeled platelets were washed with PEP buffer (10 min, 800 g) and adjusted to a concentration of 1.5 × 10^8^/mL. The remaining whole blood fraction was used for phagocytosis assay (see below).

### Sensitization of pHrodo-labeled platelets by antibodies

In brief, 500 µL aliquots of pHrodo-labeled platelets (4.5 × 10^7^ platelets) were incubated with mAbs at different concentrations (0.01, 0.1, 1, or 10 µg/mL) or serum samples (final dilution 1:10) in polypropylene tubes (Sarstedt, Nümbrecht, Germany) for 30 min in the dark. IgG isotypes were run in parallel as a control. After being washed twice with 1 mL of PEP buffer (10 min, 800 g), the platelets were resuspended in 200 µL of Hank’s Balanced Salt Solution (HBSS) containing calcium and magnesium (Anprotec) and 10 mM HEPES. A 10 µL aliquot of sensitized platelets was tested by flow cytometry to determine antibody binding. In some experiments, 250 µL aliquots of pHrodo-labeled platelets (3.75 × 10^7^ platelets) were incubated with anti-HPA-1a (NIBSC 03/152) or AB serum (final dilution 1:16) for 30 min at room temperature in the dark and processed as described above.

### Adjustment of monocyte concentration in whole blood

Leukocyte count in whole blood was measured using a cell counter (Sysmex KX-21N, Norderstedt, Germany), and monocyte percentage in this blood fraction was determined by flow cytometry. In brief, 100 µL aliquot of whole blood fraction was incubated with 15 mL of erythrocyte lysis buffer (155 mM NH_4_Cl, 10 mM KHCO_3_, and 10 mM EDTA, pH 7.4) for 5 min under gentle shaking. After centrifugation (5 min, 450 g), the cells were washed with PBS (5 min, 450 g), resuspended in 100 µL of PBS, and incubated with 5 µL of APC-conjugated anti-CD14 mAb for 15 min in the dark. The percentage of CD14-positive monocytes in the total leukocytes was analyzed by flow cytometry (FACS Canto II, Becton Dickinson, San Jose, USA), and the absolute number of monocytes was calculated by referring to leukocyte counts (see above). Afterward, 200 µL aliquots of whole blood fraction containing 5 x 10^4^ CD14-positive monocytes were prepared for the phagocytosis of 1.5 x 10^7^ platelets (ratio: 1:300 (monocytes: platelets) to mimic physiological conditions.

### Microscopic analysis of platelet phagocytosis

Microscopic analysis of platelet phagocytosis was performed as previously described with minor modifications.^19^ In brief, 200 µL aliquots of PBMC (2 × 10^7^/mL in RPMI containing 10% FCS) were incubated in a microtiter well for 1 h at 37°C and 5% CO_2_. After 30 min, the platelets were added with 5 µL of APC-conjugated anti-CD14 antibody (see above) and 5 µL of SYTOX Blue (dilution 1:100) and incubated again for 30 min. The platelets were washed with 200 µL of warm HBSS buffer (Aprotec) for 10 min at 800 g. The adherent monocytes were added with 50 µL aliquots of HBSS buffer before being incubated with 50 µL of sensitized pHrodo-labeled platelets for 1 h at 37°C and 5% CO_2_. Platelet phagocytosis was then examined by THUNDER DMi8 Live Cell Imager (Leica, Wetzlar, Germany) at 100× magnification.

Platelet phagocytosis was also examined by electron microscopy. Adherent monocyte layers were fixed with a fixation solution (2% paraformaldehyde, 2% glutaraldehyde, and 0.02% picric acid in 0.1 M phosphate buffer) for 2 h on ice. After fixation with 1% osmium tetroxide (Carl Roth, Karlsruhe, Germany) and 0.1 M cacodylate buffer (ThermoFisher) for 30 min, the cells were washed, dehydrated, embedded in EPON resin (SERVA, Heidelberg, Germany), and polymerized. Ultrathin sections (80–90 nm) were mounted on Formvar mesh (Plano, Wetzlar, Germany) and treated with 0.5% uranyl acetate (Science Services, Munich, Germany) and 3% lead citrate solution (Sigma-Aldrich) as previously described (45). Finally, the sections were investigated and recorded in a LEO912AB transmission electron microscope (Carl Zeiss, Oberkochen, Germany), and the photos were merged with ImageJ and Adobe Photoshop 2022.

## Data availability statement

The original contributions presented in the study are included in the article/**Supplementary Material**. Further inquiries can be directed to the corresponding author.

## Ethics statement

The studies involving humans were approved by Ethics Committee of the Medical Faculty, Justus Liebig University, Giessen, Germany (file no. 82/09 and file no. 05/00). The studies were conducted in accordance with the local legislation and institutional requirements. The participants provided their written informed consent to participate in this study.

## Author contributions

PA: Formal analysis, Writing – review & editing, Investigation, Methodology. NB: Formal analysis, Investigation, Writing – review & editing, Data curation, Software. MS: Data curation, Formal analysis, Validation, Writing – review & editing. GM: Formal analysis, Investigation, Methodology, Writing – review & editing. JR: Investigation, Writing – review & editing. CK: Investigation, Writing – review & editing. NC: Resources, Writing – review & editing. DT: Methodology, Writing – review & editing. BB: Formal analysis, Writing – review & editing. GB: Data curation, Formal analysis, Funding acquisition, Resources, Software, Validation, Writing – review & editing. SS: Conceptualization, Data curation, Formal analysis, Project administration, Supervision, Validation, Visualization, Writing – original draft, Writing – review & editing.

## Supplementary material

Supplementary Figure 1 Schematic of whole blood platelet phagocytosis assay (WHOPPA). Platelets were isolated from platelet-rich plasma (PRP) derived from ACD-anticoagulated blood. After washing, the platelets were labeled with a fluorescent dye (pHrodo) and adjusted to a concentration of 10^8^/mL as indicated. After incubation with platelet antibodies, the sensitized platelets were washed and adjusted to a concentration of 1.5 x 10^7^ in 200 µL. The remaining blood (ACD-anticoagulated blood minus PRP) was washed once, and the monocytes were adjusted to a concentration of 25 x 10^4^/200 μL. In some experiments, the blood was incubated with mAbs against FcγRs, and the sensitized pHrodo-labeled platelets (5 x 10^4^/200 μl) and monocytes (1.5 x 10^7^/200 μL) were mixed. The percentage of neutrophils and monocytes engulfing platelets was determined by flow cytometry.

Supplementary Figure 2 Microscopic analysis of platelet phagocytosis. *Left)* by fluorescence microscopy and *right)* by electron microscopy. DB, Dense Bodies; DTS, Dense Tubular System; OCS, Open Canicular System (see methods **Supplementary Data**).

Supplementary Figure 3 Comparison of pHrodo positive and high positive monocyte subsets. Flow cytometry analysis of human monocyte subsets after incubating the whole blood with AB serum or anti-HPA-1a opsonized pHrodo-labeled platelets as indicated. Whole blood leukocytes were gated for classical (CD14^++^CD16^−^), intermediate (CD14^++^CD16^+^), nonclassical (CD14^+^CD16^++^), and shifted (CD14^+^CD16^−^) monocytes. **(A)** Overlay of pHrodo negative (blue) with pHrodo positive (red) population and **(B)** pHrodo high population. Figure is representative of five biological replicates (n = 5).

Supplementary Figure 4 Monocyte subset and pHrodo signal intensity. Flow cytometry analysis of human monocyte subsets for pHrodo after the whole blood was incubated with AB serum or anti-HPA-1a opsonized pHrodo-labeled platelets as indicated. Whole blood leukocytes were gated for classical (CD14^++^CD16^−^), intermediate (CD14^++^CD16^+^), nonclassical (CD14^+^CD16^++^), and shifted (CD14^+^CD16^−^) monocytes. PHrodo signal intensity was analyzed by gating monocyte subsets for pHrodo negative (blue) and positive (red) populations. Figure is representative of five biological replicates (n = 5).

Supplementary Figure 5 Analysis of native and deglycosylated mAb against HPA-1a using silver staining and immunoblotting. Purified deglycosylated IgG (lanes 1 and 3) and native IgG (lanes 2 and 4) were analyzed by 7.5% SDS-PAGE under reducing conditions. After being transferred onto the polyvinylidene difluoride membrane, the proteins were stained with lens culinaris lectin using the biotin-avidin/streptavidin detection system.
