# Supplementary material for: In vitro analysis of anti-HPA-1a dependent platelet phagocytosis and its inhibition using a new whole blood phagocytosis assay (WHOPPA)
Source: Front Immunol. 2023 Nov 21;14:1283704. doi: 10.3389/fimmu.2023.1283704 (PMC10702767; doi:10.3389/fimmu.2023.1283704)
Supplement: Supplementary Figure 1 — Schematic of whole blood platelet phagocytosis assay (WHOPPA). Platelets were isolated from platelet-rich plasma (PRP) derived from ACD-anticoagulated blood. After washing, the platelets were labeled with a fluorescent dye (pHrodo) and adjusted to a concentration of 108/mL as indicated. After incubation with platelet antibodies, the sensitized platelets were washed and adjusted to a concentration of 1.5 x 10^7^ in 200 µL. The remaining blood (ACD-anticoagulated blood minus PRP) was washed once, and the monocytes were adjusted to a concentration of 25 x 104/200 μL. In some experiments, the blood was incubated with mAbs against FcγRs, and the sensitized pHrodo-labeled platelets (5 x 104/200 μl) and monocytes (1.5 x 107/200 μL) were mixed. The percentage of neutrophils and monocytes engulfing platelets was determined by flow cytometry. [file DataSheet_1.pdf]

Table 1S: **List of sera containing anti-HPA-1a antibodies used in this study**
The reactivities of the maternal sera against HPA-1a and HLA class I with paternal platelets in the MAIPA assay are presented.

Number	Serum sample	Anti-HPA-1a (OD)	Anti-HLA Class I (OD)
1	61853/19	0.165	n.d
2	46771/15	2.653	n.d
3	56843/18	1.913	n.d
4	43086/14	2.937	n.d
5	62921/19	2.909	n.d
6	43277/14	1.170	n.d
7	53860/17	1.582	n.d

Table 2S

CD16	Fluorochrome	Clone	IgG Subclass	Company
CD1c	AF700	L161	IgG1κ	Biolegend
CD3	AF700	HIT3a	IgG2aκ	Biolegend
CD14	APC	M5E2	IgG2aκ	Biolegend
CD14	PE-Dazzle 594	M5E2	IgG2aκ	Biolegend
CD14	BV605	M5E2	IgG2aκ	Biolegend
CD16	FITC	LNK16	IgG1	Biorad
CD19	AF700	HIB19	IgG1κ	Biolegend
CD32	APC-Fire750	FUN-2	IgG2bκ	Biolegend
CD32B/C	AF647	S180005H	IgG1κ	Biolegend
CD45	BV510	HI30	IgG1κ	Biolegend
CD56	BV605	5.1H11	IgG1κ	Biolegend
CD64	BV421	10.1	IgG1κ	Biolegend
CD66b	PE/Cy7	QA17A51	IgG1κ	Biolegend
HLA-DR	PerCP/Cy5.5	Tü36	IgG2bκ	Biolegend
Isotype-1	FITC	MOPC-21	IgG1κ	Biolegend
Isotype-2	AF647	MOPC-21	IgG1κ	Biolegend
Isotype-3	BV421	MOPC-21	IgG1κ	Biolegend
Isotype-4	APC	MG2a-53	IgG2aκ	Biolegend
Isotype-5	PerCP/Cy5.5	MPC-11	IgG2bκ	Biolegend
Isotype-6	APC-Fire750	MPC-11	IgG2bκ	Biolegend
LIVE/DEATH Fixable Scarlet Viability Kit				Biolegend

Figure 1S

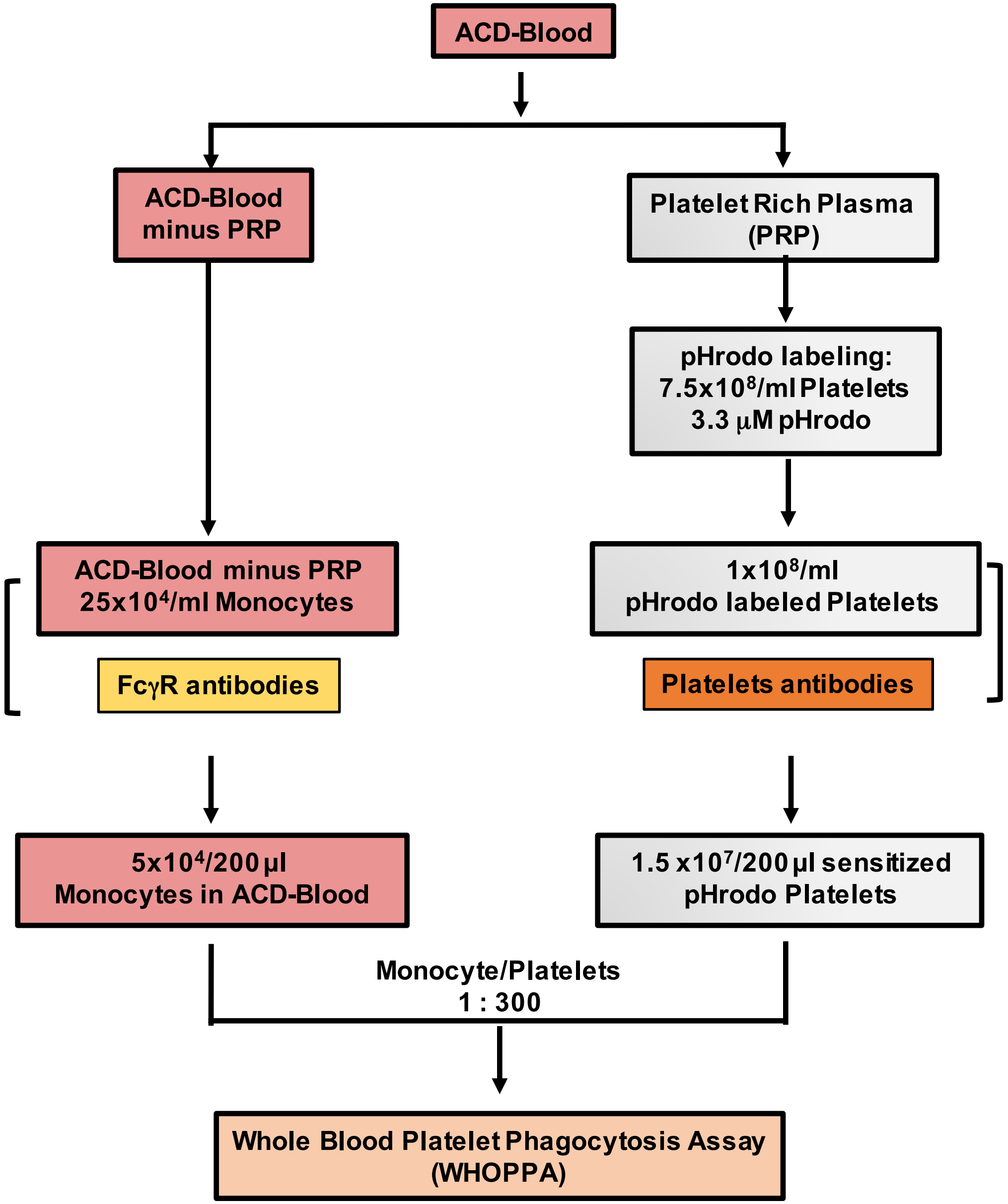

ACD-Blood
ACD-Blood minus PRP
Platelet Rich Plasma (PRP)
pHrodo labeling: 7.5x10⁸/ml Platelets 3.3 µM pHrodo
ACD-Blood minus PRP 25x10⁴/ml Monocytes
FcγR antibodies
1x10⁸/ml pHrodo labeled Platelets
Platelets antibodies
5x10⁴/200 µl Monocytes in ACD-Blood
1.5 x10⁷/200 µl sensitized pHrodo Platelets
Monocyte/Platelets 1 : 300
Whole Blood Platelet Phagocytosis Assay (WHOPPA)

Figure 2S

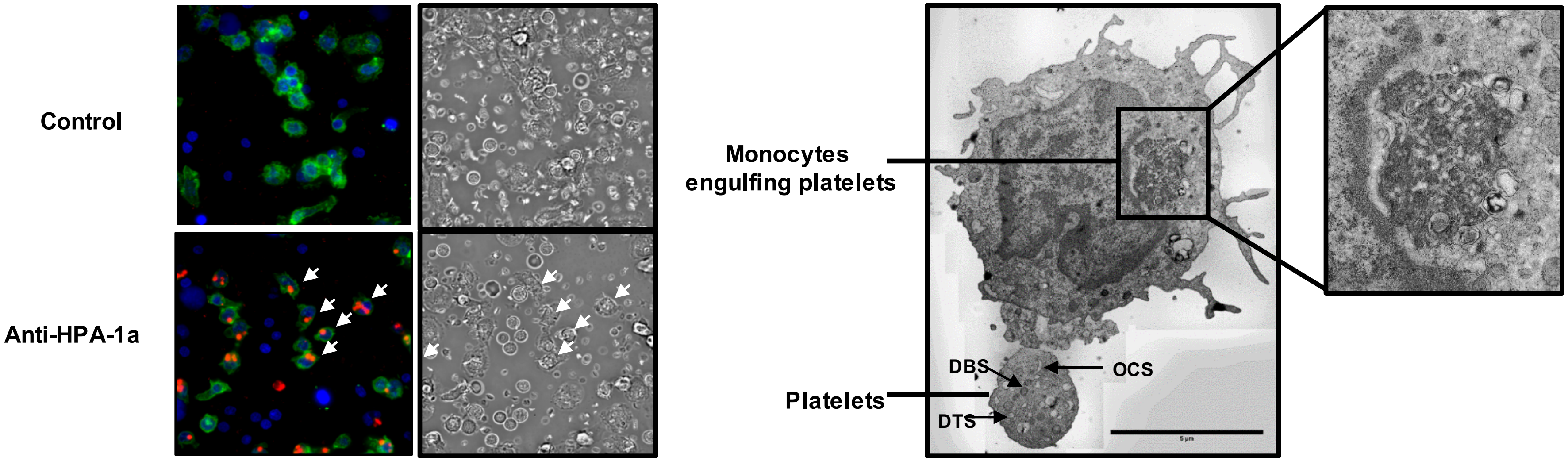

Control
Anti-HPA-1a
Monocytes engulfing platelets
Platelets
DBS
OCS
DTS

Figure 3S

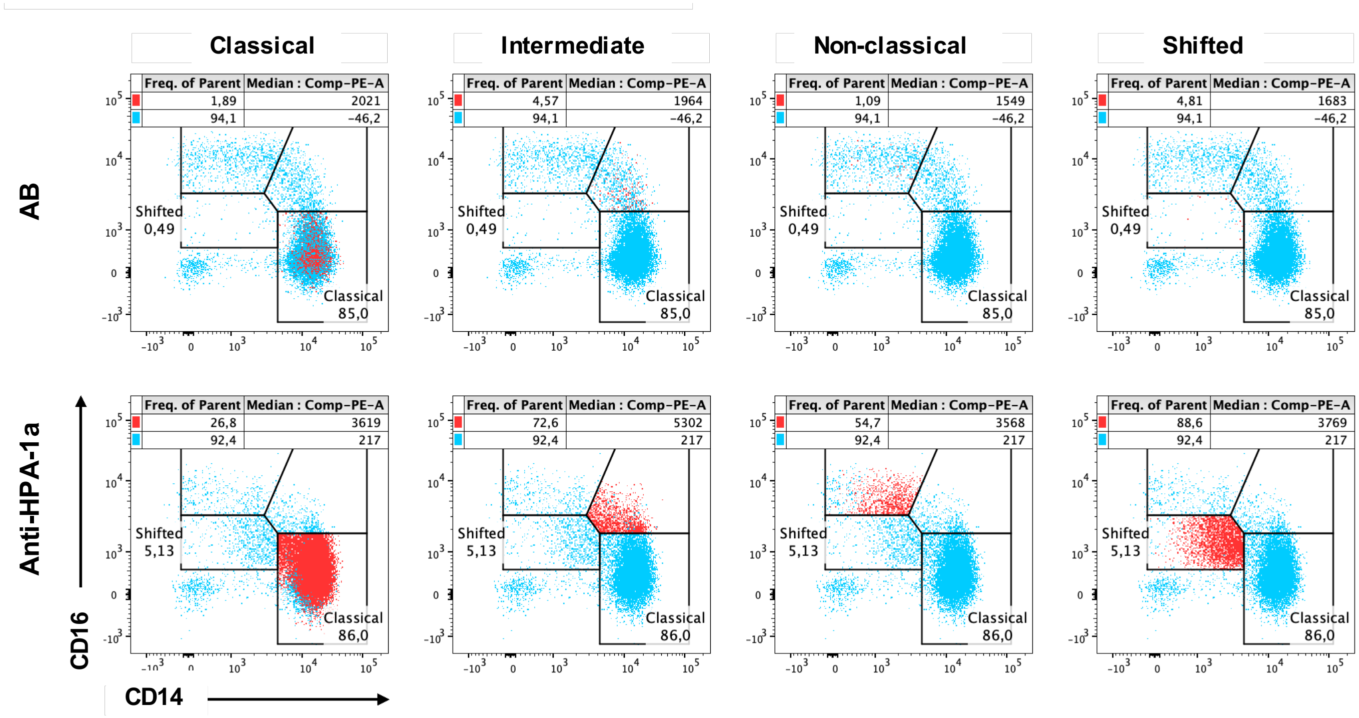

Classical
Intermediate
Non-classical
Shifted
AB
Anti-HPA-1a
CD16
CD14
Freq. of Parent | Median : Comp-PE-A
1,89 2021
94,1 -46,2
4,57 1964
94,1 -46,2
1,09 1549
94,1 -46,2
4,81 1683
94,1 -46,2
26,8 3619
92,4 217
72,6 5302
92,4 217
54,7 3568
92,4 217
88,6 3769
92,4 217
Shifted 0,49
Classical 85,0
Shifted 5,13
Classical 86,0

Figure 4S

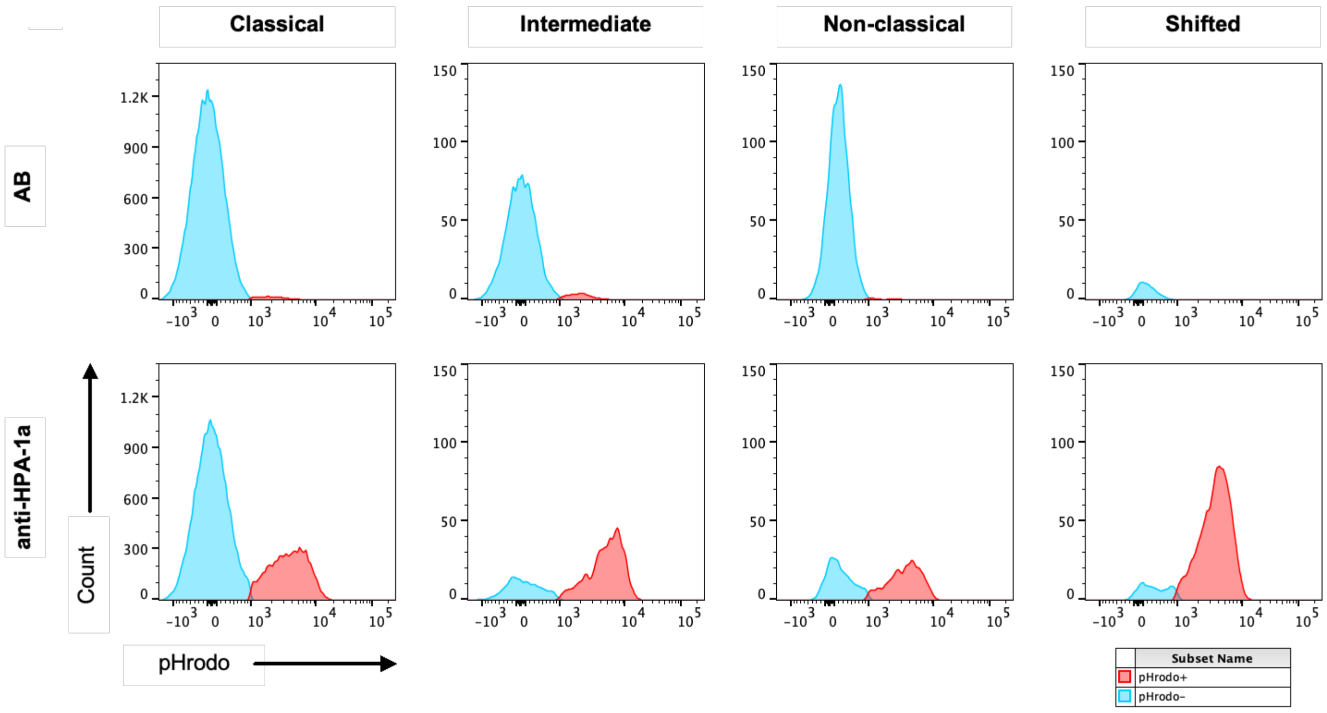
Classical
Intermediate
Non-classical
Shifted
AB
anti-HPA-1a
Count
pHrodo
Subset Name
pHrodo+
pHrodo−

Figure 5S

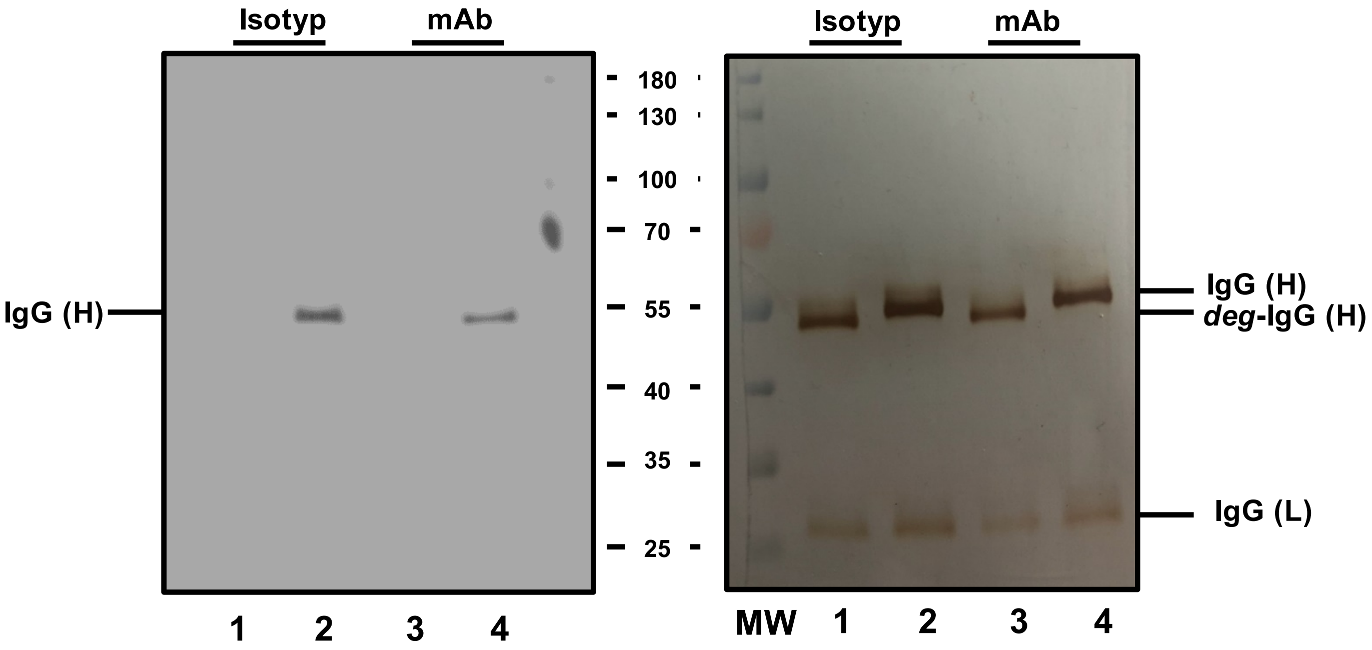

Isotyp
mAb
IgG (H)
180
130
100
70
55
40
35
25
1
2
3
4
Isotyp
mAb
IgG (H)
deg-IgG (H)
IgG (L)
MW
1
2
3
4
